# Optimization of Zinc and Aluminum Hydroxyquinolines for Applications as Semiconductors in Molecular Electronics

**DOI:** 10.3390/molecules30091896

**Published:** 2025-04-24

**Authors:** María Elena Sánchez Vergara, Francisco Iñaki Díaz Morales, Bertha Molina, Edgar Alvarez-Zauco, Lourdes Bazán-Díaz, Roberto Salcedo

**Affiliations:** 1 Facultad de Ingeniería, Universidad Anáhuac, Avenida Universidad Anáhuac 46, Col. Lomas Anáhuac, Huixquilucan 52786, Estado de México, Mexico; francisco.diaz15@anahuac.mx; 2 Universidad Politécnica de Cuautitlán Izcalli, Av. Lago de Guadalupe, Colonia Lomas de San Francisco Tepojaco, Cuautitlán Izcalli 54720, Estado de México, Mexico; 3 Facultad de Ciencias, Universidad Nacional Autónoma de México, Circuito Exterior s/n, Ciudad Universitaria, Coyoacán, Ciudad de México 04510, Mexico; mlnbrt@ciencias.unam.mx (B.M.); ezauco@ciencias.unam.mx (E.A.-Z.); 4 Instituto de Investigaciones en Materiales, Universidad Nacional Autónoma de México, Circuito Exterior s/n, Ciudad Universitaria, Coyoacán, Ciudad de México 04510, Mexico; bazanlulu@materiales.unam.mx (L.B.-D.); salcevitch@gmail.com (R.S.)

**Keywords:** metal quinoline, dispersed heterojunction, B3PW91/6-31G** calculations, organic semiconductor film, optical properties, electrical behavior

## Abstract

This work explores the dispersed heterojunction of tris-(8-hydroxyquinoline) aluminum (AlQ_3_) and 8-hydroxyquinoline zinc (ZnQ_2_) with tetracyanoquinodimethane (TCNQ) and 2,6-diaminoanthraquinone (DAAq). Thin films of these organic semiconductors were deposited and analyzed, with their structures calculated with the B3PW91/6-31G** method. The optimized structure for AlQ_3_-TCNQ, AlQ_3_-DAAq, is achieved by means of three hydrogen bonds, whereas for ZnQ_2_-DAAq, two hydrogen interactions are predicted. These structures were recalculated including the GD3 dispersion term. A stable ordering was also achieved for AlQ_3_-TCNQ-GD3, AlQ_3_-DAAq-GD3, and ZnQ_2_-DAAq-GD3 with four and two hydrogen contacts for the former and the two latter, respectively. Infrared (IR) and UV-visible spectroscopy confirmed these theoretical predictions, in addition to obtaining the optical band gap for the films. The optical band gap values ranged between 1.62 and 2.97 eV (theoretical) and between 2.46 and 2.87 eV (experimental). Additional optical parameters and electrical behavior were obtained, which indicates the potential of the films to be used as organic semiconductors. All three films showed transmittance above 76%, which also broadens the range of applications in electrodes, transparent transistors, or photovoltaic cells. Devices fabricated using these materials displayed ohmic electrical behavior, with peak current values between 2 × 10^−3^ and 6 × 10^−3^ A.

## 1. Introduction

Organic semiconductors (OS) are increasingly recognized as promising materials for the future of electronic technology and are distinguished from their inorganic counterparts due to their ecological advantages [1], chemical tunability [2], synthesis from impurity-free renewable materials [3], solution processability [4,5], low density, flexibility [1,2,3,4,5], and low-cost processing [3,5]. Furthermore, most OS have tunable optoelectronic properties, such as light absorption and emission [3], as well as the mobility of their charge carriers [5]. Recently, these properties have evolved due to the rapid development of these materials [6], allowing novel applications [1], such as their use in flexible displays [7], spintronic devices [8], organic solar cells [9], photodetectors, and organic transistors [10], to mention a few. The remarkable features of OS come from (i) their weak molecular interactions [11] due to van-der-Waals bonds, which can also make their bandwidth smaller than that of inorganic materials [1], and (ii) the anisotropic shape of their π-conjugated molecules [12]. With this anisotropy, the intermolecular arrangement and macroscopic order predispose the transport of their charge carriers and their linear or nonlinear optical responses [13,14]. Moreover, this structure allows OS to have degrees of freedom in discernible directions, which, by controlling the molecular orientation at a macroscopic scale, enhances the performance of devices fabricated with them [15].

The anisotropic OS can contain the 8-hydroxyquinolines (8HQs), a family of lipophilic metal ion chelator compounds [16]. Structurally, the 8HQs are bicyclical compounds that incorporate a pyridine ring linked to a phenol. The hydroxyl group at position 8 is near the heterocyclic nitrogen, making 8HQs excellent ligands for metal ions, forming complexes in which the ions are bound in the central position and are associated with the molecule by 4 to 6 covalently coordinated bonds. Out of the most common metal ions, those relevant to this work are Al^3+^, Zn^2+^, Cu^2+^, and Mg^2+^ [17]. Mainly, 8HQs have been used as preservatives in the textile industry, in the formation of metal–metal complexes and as anti-HIV agents in medicine [18]. Notably, when bound to metal ions, such as metal quinolines (MQs), they have applications in optoelectronic devices, namely organic light-emitting diodes (OLEDs) [19], organic field-effect transistors (OFETs), sensors [20], and as complements to inorganic materials in photovoltaic cells, optical waveguides, and lasers [21]. Furthermore, due to their excellent thermal stability, efficient luminescence, and charge transport properties, they are of great interest in research applications for molecular electronics [20,21].

This study aims to analyze the effect of the dispersed heterojunction in the optoelectronic properties of the 8-tris-(8-hydroxyquinoline) aluminum (AlQ_3_) and 8-hydroxyquinoline zinc (ZnQ_2_) metal complexes. AlQ_3_ is formed by the union between an aluminum atom located in the central position, and three HQ ligands, through bidentate bonds [22,23]. ZnQ_2_ is built in a similar way, with the zinc atom being linked by bidentate bonds to two HQ ligands. Because of their chemical stability, both complexes have been extensively studied for their luminescence, photoconductivity, field emission, and charge transport properties [22,24]. These properties are dependent on the metal ion, its crystalline structures, state of aggregation, and intramolecular interactions [22]. Regarding interactions, AlQ_3_ and ZnQ_2_ have been underexplored as far as their intermolecular interactions with other molecules that have electrical properties and can increase their behavior as OS and lead to more efficient organic devices. In optoelectronic and photovoltaic devices, the bulk or dispersed heterojunction structure can be used, in which the active layer is formed by a homogeneous mixture of electron donor and acceptor OS. This mixture provides a larger contact surface between the two materials, which improves exciton dissociation efficiency and, consequently, generates more electrical current [25]. In this study, both MQs were mixed with 7,7,8,8-tetracyanoquinodimethane (TCNQ) and 2,6-diaminoanthraquinone (DAAq) to assess their properties and behavior as potential as dispersed heterojunction films. TCNQ is a compound which has a particular structure in such a way that it possesses low dimensionality or anisotropy and forms energy bands [26]. Its disposition to accept electrons allows it to form charge transfer complexes, with various electron donor molecules, metal ions [26,27] and polycyclic aromatic compounds [28]. It is thus possible to apply it in photovoltaic devices, OLEDs [29], and various optoelectronic applications [30]. Like hydroxyquinolines, TCNQ has not been studied in enough detail as a component of dispersed heterojunction films. This work deepens the study in this area. The study of DAAq as a component of dispersed heterojunctions also requires more advancement. It is a compound that can donate electrons thanks to its amino groups and can accept electrons due to the anthraquinones in its structure. DAAq belongs to the family of anthraquinones, which, because of their aromatic skeleton, are rigid and allow easy substitution reactions [31]. These compounds have been studied for their potential applications as cathodes in metal-ion batteries [32,33], and they are commonly used in biosensors by virtue of their redox properties [33,34]. In this work, AlQ_3_ and ZnQ_2_ were employed to prepare films with dispersed heterojunction architecture with TCNQ and DAAq. The films were characterized in their morphology and, subsequently, in their optical behavior. As an important complement to the study, a hybrid method was applied, combining Becke’s gradient corrections for exchange with Perdew–Wang’s correlation corrections. This approach allowed for a comparison of both the band gap and the optical behavior of the OS, which were ultimately used in the fabrication of single-layer devices. These devices were then evaluated for their electrical performance. The novelty of this work lies in the fact that, in optoelectronics, metallic hydroxyquinolines have primarily been studied in their pristine form. Here, however, the focus is on their role as components in dispersed heterojunctions.

## 2. Materials and Methods

### 2.1. Fabrication of Dispersed Heterojunction Films

The reagents employed, tris-(8-hydroxyquinoline) aluminum (AlQ_3_: C_27_H_18_AlN_3_O_3_), 8-hydroxyquinoline zinc (ZnQ_2_: C_18_H_12_N_2_O_2_Zn), 7,7,8,8-tetracyanoquinodimethane (TCNQ: C_12_H_4_N_4_), and 2,6-diaminoanthraquinone (DAAq: C_14_H_10_N_2_O_2_), were obtained from commercial sources (Sigma-Aldrich, Carlsbad, CA, USA). Further purification was not required for their use. The AlQ_3_-TCNQ, AlQ_3_-DAAq, and ZnQ_2_-DAAq blends were prepared in a 2:1 weight ratio [25,35] with 200 mg of AlQ_3_ or ZnQ_2_ and 100 mg of TCNQ or DAAq in 5 mg/mL acetonitrile (C_2_H_3_N) solution. All solutions were refluxed for 24 h, filtered, and dried under vacuum to remove any non-dissolved material. The disperse heterojunction was analyzed by IR spectroscopy in KBr pellets, using a Nicolet iS5 FTIR spectrometer (Thermo Fisher Scientific Inc., Waltham, MA, USA). The assessment of the thermal stability of the samples was carried out with NETZSCH equipment (Erich Netzsch GmbH & Co. KG., Selb, Germany) model Jupiter STA 449 C with simultaneous TGA-DSC analysis. They were performed from room temperature up until 850 °C, at a heating rate of 10 °C/min, in an extra dry air atmosphere. An extra dry air atmosphere was used instead of an inert atmosphere because compounds with DAAq remain stable below 300 °C in an Ar and Air atmosphere [36]. Considering this and the fact that the generated compounds are considered organic semiconductors, it is important to perform the thermal analysis under the operating conditions for possible applications. To ensure uniformity, it was decided to perform TGA-DSC in extra dry air for all synthesized compounds. High-resolution imaging and chemical analysis were performed using a high-resolution transmission electron microscope (HR-TEM) JEOL ARM-200F (JEOL DE MEXICO S.A. DE C.V., CDMX, Mexico) operated at 200 kV. The films were deposited by sublimation in a high vacuum chamber (Intercovamex, S.A. de C.V., Cuernavaca, Morelos, Mexico) consisting of a mechanical pump with an initial vacuum of 10^−3^ Torr and a turbo molecular pump that produced a final vacuum of 10^−5^ Torr. The materials were heated to 300 °C to carry out thin film deposition at a rate of 1.4 Å/s for AlQ_3_-TCNQ, 0.4 Å/s for AlQ_3_-DAAq, and 11.4 Å/s for ZnQ_2_-DAAq. The deposition speed obtained depended on the composition of each film, and the sum of the molecular weight of their components. Molecular weights vary according to ZnQ_2_-DAAq < AlQ_3_-TCNQ < AlQ_3_-DAAq. The energy required is inversely proportional to the rate to evaporate and deposit the species with lower molecular weight. Thin films were deposited on glass, n-type silicon, and on indium tin oxide-coated polyethylene terephthalate (PET-ITO). The films on n-type silicon were analyzed by IR spectroscopy to determine whether the components of the films were chemically degraded during deposition. UV-vis spectroscopy of the films on glass was performed using a Unicam Spectrophotometer model UV300 (Thermo Fisher Scientific Inc., Waltham, MA, USA). The film’s morphological features, thickness, roughness, and micromechanical properties were investigated in contact mode on n-type silicon. With a Nanosurf Naio atomic force microscope (Nanosurf AG, Liesta, Erlen, Switzerland) with an NTEGRA platform and Gwyddion 2.66 software. Thin films deposited on PET-ITO allowed the fabrication of flexible devices using ITO as an anode and Ag as a cathode. These single-layer devices were characterized in their electrical behavior, making use of a sensitization station with a Next Robotics lighting controller circuit (Comercializadora K Mox, S.A. de C.V., CDMX, Mexico), a programmable voltage source, and a Keithley 4200-SCS-PK1 automatic range picoammeter (Tektronix Inc., Beaverton, OR, USA).

### 2.2. Computational Method

All hydroxyquinolines were optimized by the application of a hybrid method based on the combination of Becke’s gradient corrections [37] for exchange and Perdew–Wang’s correlation [38]. This is the scheme for the B3PW91 method, which is included in the Gaussian16 [39] package. The calculations were conducted using the 6-31G** basis set; it is important to highlight that this method was chosen due to its strong performance in predicting structural parameters [40]. Likewise, the Perdew–Burke–Ernzerhof (PBE) [41] generalized gradient approximation (GGA) function was used for the exchange and correlation terms in the Kohn–Sham Hamiltonian, as implemented in the Amsterdam Density Functional (ADF2013.01) [42] package. This function was chosen because it aligned very well with experimental vibrational and optical parameters for organometallic compounds [43]. The standard Slater-type orbital basis set was used, with a quality of triple-ζ, plus two polarization functions (TZP). For the self-consistent field (SCF) convergence, an accuracy of 10^−5^ Hartree and a gradient of 10^−4^ Hartree/Å were selected. In all cases, frequency calculations were carried out at the same level of theory used for the geometrical calculation to confirm that the optimized structures were at a minimum on the potential energy surface. The 200 lowest allowed singlet-singlet transitions were calculated in order to simulate the absorption spectra for each structure. The Hirshfeld method [44] was applied to evaluate the nature of hydrogen bonds. Although this method is known as a crystal interaction tool, it has been applied to discrete molecules [45,46] with successful results and, in the present case, was used in the same way.

## 3. Results and Discussion

### Optimization of Hydroxyquinolines

This study builds upon our previous research [47], where calculations were performed on ZnQ_2_ and TCNQ molecules. In this work, we compare the new results with those obtained in that earlier study to complement and extend the current investigation. A key finding, once again, is that hydrogen bonds play a fundamental role in the formation of OS with optoelectronic behavior. The development of new OS that enhance the applications of silicon and its derivatives remains essential in the field of molecular electronics.

**Figure 1:**
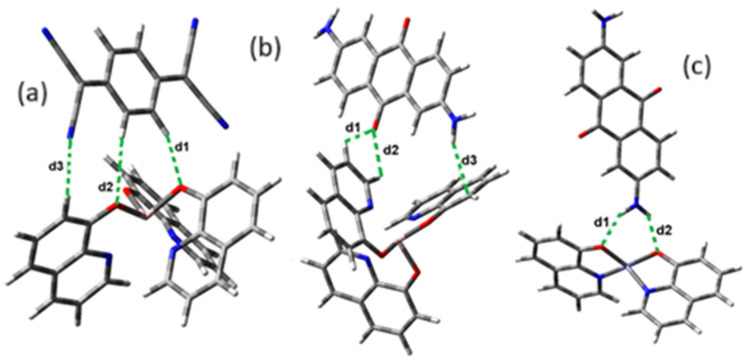
Schematization of the optimized structure of (**a**) AlQ_3_-TCNQ, (**b**) AlQ_3_-DAAq, and (**c**) ZnQ_2_-DAAq using the B3PW91/6-31G** method.

**Figure 2:**
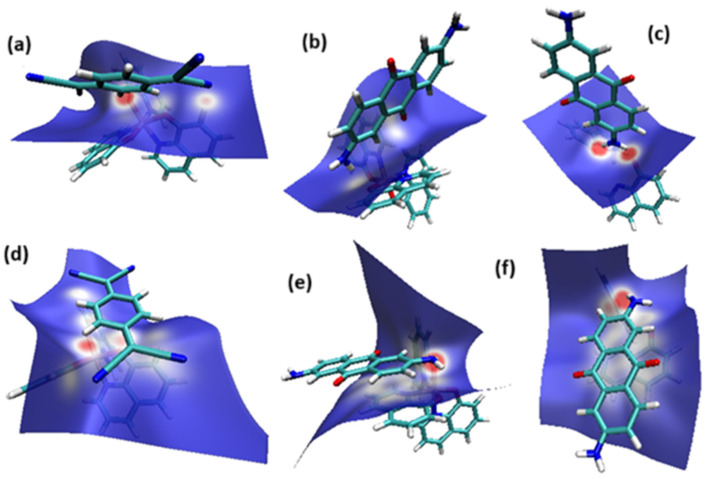
Hirshfeld Surface for (**a**) AlQ_3_-TCNQ, (**b**) AlQ_3_-DAAq, (**c**) ZnQ_2_-DAAq, (**d**) AlQ_3_-TCNQ-GD3, (**e**) AlQ_3_-DAAq-GD3, and (**f**) ZnQ_2_-DAAq-GD3.

The optimized structure obtained for AlQ_3_-TCNQ, AlQ_3_-DAAq, and ZnQ_2_-DAAq using method B3PW91/6-31G** are shown in Figure 1, where the hydrogen bonds are also highlighted. Note that for the first two, the equilibrium structures are reached by means of three hydrogen bonds (see Figure 1a,b) with lengths of 2.20, 2.52, and 2.71 Å for AlQ_3_-TCNQ and 2.62, 2.64, and 2.73 Å for AlQ_3_-DAAq. For ZnQ_2_-DAAq, only two hydrogen interactions are predicted with lengths of 2.07 and 2.12 Å. Clearly, the last two correspond to the strongest interactions. This can be confirmed by visualizing the Hirshfeld surface mapped with d_norm_. Figure 2 illustrates the Hirshfeld surfaces for AlQ_3_-TCNQ, AlQ_3_-DAAq, and ZnQ_2_-DAAq, where blue, white, and red regions indicate negligible, moderate, and high electron density, respectively. In AlQ_3_-TCNQ, two hydrogen bonds between Al-O and H-C groups can be clearly identified—one strong and one moderate—corresponding to the red and white zones in Figure 2a. Additionally, a third interaction, C-H···N=C, is detected, which can be classified as a moderate hydrogen bond due to its weak reddish shades. For AlQ_3_-DAAq, only three bright white zones are observed (Figure 2b), two of which correspond to moderate hydrogen bonds between C-H and O=C species. The third interaction is established between a carbon atom in the quinoline ring and an H-N group of the DAAq. Finally, in ZnQ_2_-DAAq (Figure 1c), two strong hydrogen bonds are easily identified by the presence of two intense red points on its Hirshfeld surface. These are associated with Zn-O···H-N hydrogen contacts, highlighting their significant role in molecular interactions (Figure 2c).

**Figure 3:**
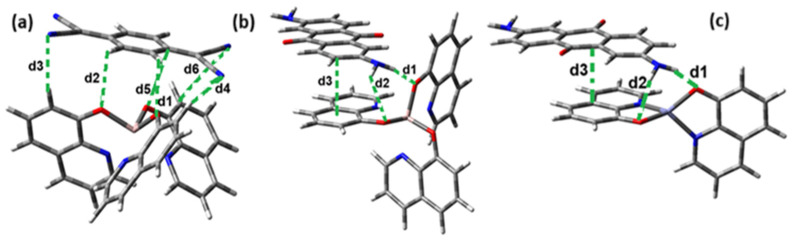
Hirshfeld schematization of the optimized structure of (**a**) AlQ_3_-TCNQ-GD3, (**b**) AlQ_3_-DAAq-GD3, and (**c**) ZnQ_2_-DAAq-GD3 using the B3PW91-GD3/6-31G** method.

The interaction energies have been calculated to assess the strength of the exchange; this feature was estimated as the difference between the energy of the stable arrangement obtained for AlQ_3_-TCNQ, AlQ_3_-DAAq, and ZnQ_2_-DAAq and energies of isolated species [48]. Despite the previous results for distances and Hirshfeld surfaces, the interaction energies obtained are relatively small (−7.5, −4.9, and −8.1 kcal/mol for AlQ_3_-TCNQ, AlQ_3_-DAAq, and ZnQ_2_-DAAq, respectively), suggesting that the strength of the interactions in these compounds are in the order of a weak hydrogen bond. The Wiberg bond index (WBI) reinforces these results, since for AlQ_3_-TCNQ, the total WBI is 0.025, whereas for AlQ_3_-DAAq, it is only 0.011 (see Table 1). According to the WBI, in ZnQ_2_-DAAq, the two strongest hydrogen contacts can be found, with WBIs of 0.024 and 0.021. Based on these results, we proceeded with a new optimization process for these structures, incorporating the dispersion term GD3 into the corresponding Hamiltonian. As shown in Figure 3, the inclusion of GD3 in the calculations led to different stable arrangements compared to those obtained without it. In these newly optimized structures, we identified multiple hydrogen contacts and instances of eclipsed stacking. Upon initial inspection, four hydrogen contacts with an average length of 2.64 Å were recorded for AlQ_3_-TCNQ-GD3, while AlQ_3_-DAAq-GD3 and ZnQ_2_-DAAq-GD3 each exhibited only two, measuring 1.90 and 3.03 Å for the former and 2.00 and 2.74 Å for the latter (see Table 2). However, Hirshfeld surface analysis reveals a different perspective: in AlQ_3_-TCNQ-GD3, seven contacts between TCNQ and tris-(hydroxyquinoline) aluminum (Figure 3 and Table 2) can be identified. Among them, two hydrogen interactions between C-H and O-Al groups appear as red points in Figure 2d. A third red zone corresponds to an interaction between an O-Al group and a carbon atom in the ring. The remaining four interactions, visualized as white regions, indicate moderate interactions corresponding to two hydrogen bonds (C+N···H-C), one dihydrogen interaction, and one C=N···C contact (Figure 3 and Table 2).

For AlQ_3_-DAAq-GD3 and ZnQ_2_-DAAq-GD3, the Hirshfeld surfaces each exhibit an intense red spot, highlighting the strongest interaction between NH- and O-Al groups. Additionally, both structures show eclipsed stacking and the presence of delocalized electrons, which can be identified through the extended white zones in Figure 2e,f, forming a tenuous ring. The distance d3d_3 between two adjacent rings—one belonging to DAAq and the other to hydroxyquinoline—is approximately 3.26 Å, comparable to the spacing between graphitic layers.

The interaction energies for AlQ_3_-TCNQ-GD3, AlQ_3_-DAAq-GD3, and ZnQ_2_-DAAq-GD3 are −22.9, −27.9, and −27.8 kcal/mol, respectively, accounting for all interactions between both species in each arrangement. Table 2 indicates that the WBI values are relatively low for each atomic pair. However, for AlQ_3_-TCNQ-GD3, up to seven contacts were found between both molecules, resulting in a total WBI of 0.031. The strong hydrogen bond identified in the Hirshfeld surface for AlQ_3_-DAAq-GD3 is confirmed by a WBI of 0.040. In contrast, for ZnQ_2_-DAAq-GD3, one of the two hydrogen contacts appears weaker, while the other has slightly strengthened, yielding a WBI of 0.026.

**Figure 4:**
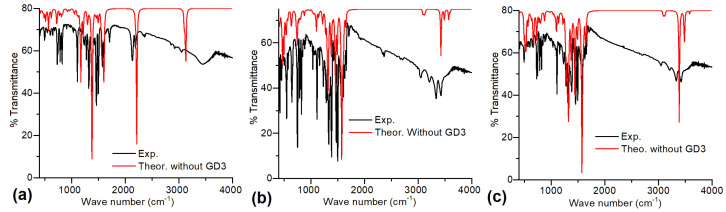
Comparison between experiments in KBr pellets and theoretical IR spectra for (**a**) AlQ_3_-TCNQ, (**b**) AlQ_3_-DAAq, and (**c**) ZnQ_2_-DAAq.

As in our previous work, the theoretical B3PW91 and PBE spectra only differ by a small shift in the bands. This comparison is essential because, according to the previous results and according to the comparison with the IR spectra of the precursor compounds (see Table 3), it is possible to carry out the interaction between AlQ_3_ and ZnQ_2_ with TCNQ and DAAq. The theoretical IR spectroscopy results must be compared with the experimental data obtained from IR spectroscopy in KBr pellets, focusing on the precursor compounds and the AlQ_3_-TCNQ, AlQ_3_-DAAq, and ZnQ_2_-DAAq systems. A comparison between the experimental IR spectra and the theoretical calculations reveals that, in general, the spectra calculated with dispersion corrections align more closely with the experimental results (see Figure 4), particularly for the AlQ_3_-TCNQ film.

Thus, we use the spectra calculated with the GD3 term as a reference for characterizing the experimental spectra. For AlQ_3_-TCNQ, the most intense peaks in the 400–600 cm^−1^ region (see Figure 4a) correspond to in- and out-of-plane deformations arising from scissoring and twisting vibrations in AlQ_3_, which, in most cases, are coupled with twisting or wagging modes in TCNQ. Additionally, the peak near 650 cm^−1^ is associated with the symmetric stretching of O-Al bonds. In the first zone of the band at 700–850 cm^−1^, we found the breathing mode of the Q_3_, whereas the next section is completely dominated by wagging vibrations in both TCNQ and Q_3_. The narrow band at 1050–1100 cm^−1^ belongs to C-N and Al-N bond stretching and scissoring of H atoms in AlQ_3_. The extended band from 1170 to 1730 cm^−1^ corresponds to C-C, C-O, and C-N stretching bonds in the hydroxyquinoline rings. As expected, the band in 2050–2250 cm is the signal for TCNQ and can be associated with C-N bond stretching of the cyano groups. Differences between the theoretical and experimental IR spectra are observed, as previously reported in our earlier work, particularly in relation to the C-H vibrations in TCNQ [47]. Notably, a greater discrepancy appears in the 3000–3650 cm^−1^ region, which corresponds to symmetric and asymmetric C-H bond stretching in both AlQ_3_ and TCNQ. For AlQ_3_-DAAq, two broad bands can be distinguished in the 400–1750 cm^−1^ region (see Figure 4b), both containing mixed vibrational modes of AlQ_3_ and DAAq. In the 400–600 cm^−1^ band, alternating scissoring and twisting modes in Q_3_ and DAAq are evident. The narrow peak around 646 cm^−1^ corresponds to asymmetric and symmetric O-Al stretching vibrations, while the breathing modes of Q_3_ are found in the first section of the 750–1000 cm^−1^ band, along with wagging motions of H atoms in both Q₃ and DAAq. As with AlQ_3_-TCNQ, stretching modes appear in a broad 1000–1750 cm^−1^ band, with the most intense signals corresponding to DAAq. Within this band, alternating pure modes of DAAq and Q_3_ related to C-C and C-N stretching, accompanied by H-atom scissoring, are observed. The central section is characterized by in-plane deformations due to C-C, C-N, and C-O stretching in Q_3_, along with similar interactions in DAAq, plus H-atom rocking. The most intense breathing modes of DAAq are also found in this region. The next section similarly features C-C, C-N, and C-O stretching in both Q_3_ and DAAq, again accompanied by H-atom scissoring, with the most intense peak corresponding to C-C stretching in DAAq. Figure 4b shows that, for AlQ₃-DAAq, the experimental and theoretical spectra are in better agreement for the most energetic peaks in the 3000–3750 cm^−1^ band. The peak around 3040 cm^−1^ corresponds to C-H asymmetric stretching in Q_3_ and DAAq, while the remaining signals relate to N-H symmetric and asymmetric stretching in DAAq. For ZnQ_2_-DAAq (see Figure 4c), the most intense peaks in the 400–600 cm^−1^ band are associated with DAAq, corresponding to alternating twisting and scissoring motions in its rings. This band also contains Zn-O asymmetric stretching vibrations and intense wagging of H atoms bonded to O atoms in ZnQ_2_. In the first section of the 680–920 cm^−1^ band, the breathing modes of Q_2_ couple with twisting motions in DAAq rings, while the next section is dominated by wagging vibrations. The narrow band at 1000–1250 cm^−1^ is linked to alternating H-atom scissoring in Q_2_ and DAAq, whereas stretching modes extend across the 1300–1750 cm^−1^ range. The initial part of this band contains alternating in-plane deformations due to C-C and C-N stretching in both molecules, along with the breathing mode of DAAq, associated with the second most intense vibration in ZnQ_2_-DAAq. The most intense vibration is found in the central section, related to C-C, C-N, and C-O stretching in Q₂, coupled with C-C stretching in DAAq. In the final part of this band, C-C, C-N, and C-O stretching in Q_2_ is observed, alongside a C=O stretching mode at the band’s endpoint. Like AlQ_3_-DAAq, ZnQ_2_-DAAq shows good agreement with experimental spectra in the 3000–3600 cm^−1^ band, with signals corresponding to C-H stretching in Q_2_ and DAAq and N-H symmetric and asymmetric stretching.

The close alignment between experimental and theoretical IR spectra demonstrates the effectiveness of both approaches—with and without dispersion corrections—in characterizing molecular vibrations. This agreement supports the validity of the proposed molecular configurations for all three OS systems. Regarding the comparison between the IR spectra of AlQ_3_-TCNQ, AlQ_3_-DAAq, and ZnQ_2_-DAAq and their precursor compounds, the characteristic frequencies are presented in Table 3. Slight shifts toward lower frequencies are observed in C-N quinoline bonds, in-plane ring deformations, and O-M bonds compared to pristine hydroxyquinolines. This may indicate the presence of hydrogen bonds previously calculated. Additionally, the frequency shifts in the C=O stretching of anthraquinone in AlQ_3_-DAAq and ZnQ_2_-DAAq, as well as the shift in the C≡N band of TCNQ in AlQ₃-TCNQ, further support this observation.

Thin films with AlQ_3_-TCNQ, AlQ_3_-DAAq, and ZnQ_2_-DAAq are deposited because the fabrication of devices with OS films, besides being more economical, generates lighter, more flexible devices. This allows for a more precise control of their optoelectronic properties. After the deposition, the films were evaluated by IR spectroscopy to determine whether, during evaporation, the components of the dispersed heterojunction suffered chemical degradation. Table 3 shows a comparison of the main vibration frequencies obtained in film and pellet form. The small variation between the values is due to residual stresses in the bonds resulting from the evaporation and deposition of AlQ_3_-TCNQ, AlQ_3_-DAAq, and ZnQ_2_-DAAq; however, there is no evidence of degradation of the film components because of their deposition. This indicates that the method for preparing dispersed heterojunction films is adequate.

**Figure 5:**
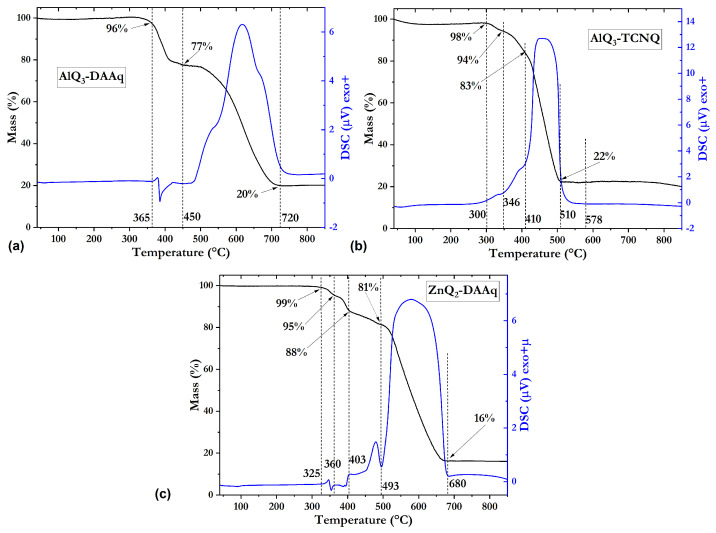
TGA and DSC curves of (**a**) AlQ_3_-DAAq, (**b**) AlQ_3_-TCNQ, and (**c**) ZnQ_2_-DAAq under an extra dry air atmosphere at a heating rate of 10°/min.

As seen in Figure 5a, the phase transition of AlQA_3_ from α-phase to γ-phase takes place at 370–385 °C, and a sharp peak with the peak temperature at approximately 385 °C is ascribed to the melting of AlQ_3_, according to a report by Wang et al. [49]. At the same interval but between 385 and 450 °C, sample AlQ_3_-DAAQ shows a mass loss around 20%, related to the decomposition of DAAQ, in good agreement to a report by Jung et al. [50]. The decomposition of AlQ_3_ was observed accompanied by melting and evaporation at 450 °C. However, between 450 and 720° C, there is mass loss; in this case, the associated evaporation temperature is around 480° C, and this value is set by the change in concavity that presents the DSC curve. The two separate behaviors are related to the weak interaction between AlQ_3_ and DAAQ compounds. Thermal analysis of the AlQ_3_-TCNQ heterojunction is presented in Figure 5b. Before 410 °C, two changes are presented; the phase transition of AlQA_3_ from α-phase to γ-phase around 375 °C and thermal decomposition of the TCNQ between 300 and 410 °C, related to the report by Usman et al. [51]. On the other hand, the decomposition of AlQ_3_ was taken from 410 to 510 °C, and the combined behaviors indicate a strong interaction of the compounds. Thermograms shown in Figure 5c present two regions. For the one before 493 °C, two phenomena occur, the melting point of ZnQ_2_ around 352 °C, which is consistent with the report by Keshmiri et al. [52] with a mass loss of 3% and possibly related to non-reacted ZnQ_2_, and the next 14% mass loss up to 493 °C related to the decomposition of DAAQ, which is also non-reacted. The second region from 493 to 662 °C is clearly related to the degradation of ZnQ_2_-DDAQ, in which case indicates a strong interaction of both components. From the above results, it is observed that the separate behaviors are related to the weak interaction of hydroxyquinoline with DAAQ and TCNQ compounds.

**Figure 6:**
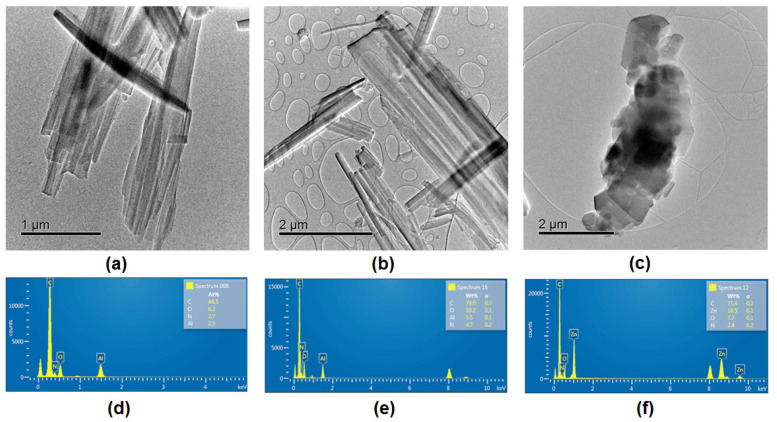
TEM images showing the morphology of the organic semiconductors: (**a**) AlQ_3_-TCNQ, displaying short nanobar structures (1–3 μm); (**b**) AlQ_3_-DAAq, exhibiting elongated nanobars (2–5 μm); and (**c**) ZnQ_2_-DAAq, consisting of nanoparticles with mixed morphologies, including hexagonal facets and semicircular shapes. EDS spectra for each respective sample (**d**–**f**).

Morphological characterization performed by transmission electron microscopy (TEM) reveals apparent structural differences in the doped metal–organic complexes. The aluminum-based complexes, AlQ₃-TCNQ (Figure 6a) and AlQ_3_-DAAq (Figure 6b), both display anisotropic, nanobar-like structures. Notably, AlQ_3_-DAAq forms considerably longer nanobars, measuring between 2 and 5 μm, while AlQ_3_-TCNQ presents shorter forms, in the range of 1 to 3 μm. This variation in morphology suggests that DAAq or TCNQ influences the anisotropic growth dynamics of the Al particle. Alternatively, ZnQ_2_-DAAq (Figure 6c) produces quasi-spherical nanoparticles with diverse morphologies, such as hexagonally faceted regions and semicircular shapes. On the other hand, in the EDS spectra of Figure 6d–f, the distribution of the elements that form the dispersed heterojunctions is observed, with the Al and Zn of the hydroxyquinolines, the O of AlQ_3_, ZnQ_2_, and DAAq, and the N that forms part of the two species of the heterojunction.

**Figure 7:**
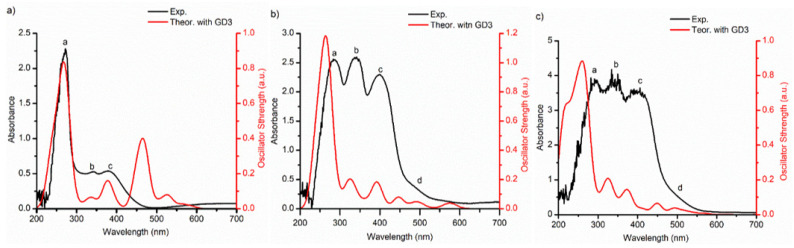
From left to right, comparison between experimental and theoretical absorbance spectra for (**a**) AlQ_3_-TCNQ-GD3, (**b**) AlQ_3_-DAAq-GD3, and (**c**) ZnQ_2_-DAAq-GD3 using TD-DFT PBE-GD3/TZP method. To build each theoretical spectrum, a Gaussian broadening of 30 nm was applied. In each spectrum the peaks and shoulders were labeled with the letters a–d.

The optical properties of AlQ_3_-TCNQ, AlQ_3_-DAAq, and ZnQ_2_-DAAq films, including absorbance, were analyzed using UV-vis spectroscopy. This analysis is crucial for understanding their behavior in organic devices, such as organic solar cells. Additionally, absorbance is highly sensitive to electronic transitions, facilitating the identification of energy bands and providing crucial insights into how AlQ_3_-TCNQ, AlQ_3_-DAAq, and ZnQ_2_-DAAq films interact with light. The experimental measurements were compared with theoretical calculations, and the results are presented in Figure 7. The absorption spectrum of the AlQ_3_-TCNQ film (Figure 7a) is dominated by a strong ultraviolet absorption band at 272 nm (band a), accompanied by two weaker bands at 342 nm (band b) and 377 nm (band c). Notably, when characterizing the experimental spectra, we observed that the calculated UV-vis spectra—including the dispersion term—align more closely with the experimental data (see Figure 7 and Table S1 in the supporting information). For this reason, the dispersion-corrected spectra were used to identify the transitions responsible for the peaks in the experimental spectra. This improved alignment may be attributed to heterogeneities or high surface roughness in thin films. Theoretically, the three characteristic absorption bands (a, b, and c) in the AlQ_3_-TCNQ-GD3 spectrum are located at 271, 340, and 376 nm, respectively (see Figure 7a and Table S1). A detailed TDDFT analysis of molecular transitions reveals that band a (272 nm experimental, 271 nm theoretical) consists of six singlet–singlet transitions. The most intense of these primarily corresponds to ligand-metal charge transfer (LMCT) and intramolecular charge transfer in hydroxyquinoline. Among the remaining transitions, those at 265.6 and 234.5 nm are also related to LMCT, while the other four can be attributed to intramolecular charge transfer in hydroxyquinoline. Regarding band b (342 nm experimental, 340 nm theoretical), the transitions HOMO → LUMO + 8 and HOMO-4 → LUMO + 2 are associated with ligand–ligand charge transfer. In contrast, band c (377 nm experimental, 376 nm theoretical) corresponds to electronic exchange between hydroxyquinoline and TCNQ species, specifically through transitions HOMO-2 → (LUMO + 5, LUMO+). The primary differences between the theoretical and experimental spectra stem from two theoretical bands corresponding to transitions at 469.7 and 527.7 nm. The absence of these bands in the experimental spectrum is likely due to intermolecular interactions within the AlQ_3_-TCNQ film.

The experimental spectrum of AlQ_3_-DAAq exhibits a broad absorption band with three prominent “fingers”—peaks a, b, and c—located at 283, 342, and 400 nm, along with a smaller shoulder, d, at 500 nm (see Figure 7b). Comparing the theoretical and experimental spectra reveals a slight blue shift in these four bands, with the predicted positions of a, b, c, and d at 265, 328, 392, and 473 nm, respectively. According to theoretical analysis, several strong transitions are identified in band a (283 nm experimental, 265 nm theoretical), six of which are detailed in Table S2. The two most intense transitions in this band correspond to ligand–ligand charge transfer (LLCT) and electronic exchange between hydroxyquinoline and DAAq species. Notably, transitions at 240.5 and 262.9 nm involve HOMO → LUMO + 17 and HOMO-2 → LUMO + 14 transitions, where electron backdonation occurs within the orbital rings of hydroxyquinoline and DAAq, while the unoccupied orbitals exhibit contributions from the metal atom. The weakest transitions in this band, as listed in Table S2, are also associated with LLCT. In band b (342 nm experimental, 328 nm theoretical), the strongest transition corresponds to intramolecular charge transfer within DAAq, while two additional transitions at 331.6 and 337.8 nm are primarily associated with intramolecular charge transfer in hydroxyquinoline. The weakest transition in this band involves charge transfer between hydroxyquinoline and DAAq species. Band c (400 nm experimental, 392 nm theoretical) is mainly associated with (HOMO-1, HOMO-2) → LUMO + 5 and HOMO-2 → LUMO + 4 transitions, which involve electronic exchange between DAAq and hydroxyquinoline. Finally, shoulder d (500 nm experimental, 473 nm theoretical) arises from two transitions: the most energetic one results from electronic exchange between hydroxyquinoline and DAAq in both directions, while the second transition is related to intramolecular charge transfer within hydroxyquinoline.

Figure 7b,c highlight the strong similarity between the experimental UV-vis spectra of AlQ_3_-DAAq and ZnQ_2_-DAAq. Like AlQ_3_-DAAq, the ZnQ_2_-DAAq spectrum features a broad absorption band with three prominent peaks (‘fingers’ a, b, and c) located at 291, 343, and 400 nm, along with a smaller shoulder, d, at 512 nm. Compared to the AlQ_3_-DAAq spectrum, the first peak exhibits a slight red shift of 8 nm, the second peak shifts by just 1 nm, the third remains unchanged, while the fourth differs by 12 nm. Additionally, the theoretical spectra of both structures share significant similarities, except for a blue shift and a pronounced shoulder at 210 nm appearing in the ZnQ_2_-DAAq spectrum.

According to theoretical results, for band a (291 nm experimental, 261 nm theoretical), five transitions have been identified (Table S3), including those associated with metal transitions. The most intense transition predominantly corresponds to intramolecular charge transfer within DAAq, with contributions from electronic exchange between DAAq and hydroxyquinoline. The second strongest transition is linked to metal–ligand charge transfer (MLCT) and intramolecular charge transfer in hydroxyquinoline. Additionally, the transition at 245.9 nm is associated with electronic exchange between DAAq and the metal center. The remaining two transitions correspond to intramolecular charge transfer in DAAq and LMCT, specifically involving HOMO-1 → (LUMO + 9, LUMO + 10), where HOMO-1 displays electron backdonation. In band b (343 nm experimental, 328 nm theoretical), the two strongest transitions primarily correspond to intramolecular charge transfer within DAAq and hydroxyquinoline, respectively, while the weakest transition is dominated by electronic exchange between DAAq and hydroxyquinoline species. Band c (400 nm experimental, 375 nm theoretical) is characterized by transitions associated with electronic exchange between hydroxyquinoline and DAAq. Meanwhile, band d (512 nm experimental, 483 nm theoretical) features intramolecular transitions within both molecules.

In the AlQ_3_-TCNQ, AlQ_3_-DAAq, and ZnQ_2_-DAAq, it is important to know the charge transport capacity that each one presents, and this is obtained from the band gap value or activation energy. Regarding the B3PW91/6-31G** and B3PW91-GD3/6-31G** calculations, the frontier molecular orbitals, HOMO and LUMO presented in Table 4, are those that determine the band gap value (ΔE). It is observed that the semiconductor AlQ_3_-TCNQ is the one with the smallest value, and therefore, the one that would have the greatest capacity for transporting electrical charges and, thus, is the best semiconductor. Incidentally, the lowest ΔE values are obtained when calculating without GD3, most likely because the position of the HOMO and LUMO molecular orbitals change when including GD3. It is also important to note that the obtained ΔE values are lower than those previously calculated and reported for pristine AlQ_3_ [19,53], which range between 3.27 and 3.9 eV, as well as those reported [47] for pristine ZnQ_2_ (3.43 eV). This suggests a positive effect of TCNQ and DAAq on hydroxyquinolines, as their presence reduces the HOMO–LUMO gap by facilitating the formation of conduction channels.

The theoretical calculations also contribute to the determination of the isosurfaces assigned to each molecular orbital. Delocalization of molecular orbitals between two isolated molecules has been studied by other groups with interesting contributions, in which orbitals as well as electronic communications are studied [54,55]. The representation of these isosurfaces in Table 4 offers an overview of the phases of the molecular orbitals, which facilitates the interpretation of the intermolecular interactions in the solid state, which is how hydroxyquinolines are found under experimental conditions when forming thin films. The structures of AlQ_3_-TCNQ, AlQ_3_-DAAq, and ZnQ_2_-DAAq are governed by relatively weak hydrogen bonds, so that the molecular interactions on which charge transport depends should be favored by the adequate overlapping of the orbitals between neighboring molecules. It is valuable to consider that according to Table 4, the position of the HOMO and LUMO orbitals changes, depending on each type of molecule but also, on the type of calculation, with or without the GD3 contribution. In the case of AlQ_3_-TCNQ, HOMO is in one of the hydroxyquinolines, and LUMO is in TCNQ. In the case of AlQ_3_-DAAq, as in the previous structure, HOMO is in one of the hydroxyquinolines, and LUMO is in DAAq. Finally, for ZnQ_2_-DAAq, the position of the molecular orbital’s changes; HOMO is in DAAq, and LUMO is in the hydroxyquinoline with a very small contribution from the metal atom. On the other hand, for the systems with a GD3 contribution, there are significant changes in the location of the orbitals with respect to the orbitals calculated without this contribution: in AlQ_3_-TCNQ-GD3, HOMO is mainly distributed in the three hydroxyquinolines but does not present a contribution from the Al atom, and LUMO is in TCNQ. In the case of AlQ_3_-DAAq-GD3, HOMO shows equivalent contributions from both hydroxyquinoline and DAAq, and no contributions from the metal are observed; LUMO is mainly concentrated on DAAq, although it also shows minor contributions from the hydroxyquinoline and a pair of lobes connecting both fragments. For ZnQ_2_-DAAq-GD3, HOMO is also localized on a hydroxyquinoline and DAAq, with a very small contribution from the Zn atom; LUMO is widely distributed on both DAAq and the hydroxyquinoline, with one lobe connecting both fragments.

Going back to the discussion on the band gap (ΔE), it appears that in AlQ₃-TCNQ and AlQ_3_-TCNQ-GD3, the localization of HOMO in hydroxyquinoline and LUMO in TCNQ enhances charge transport compared to the other molecules. This effect can be attributed to the structural characteristics and strong electron-withdrawing ability of TCNQ, which facilitates better orbital overlap and promotes charge transfer [26,27]. These findings should be further validated through experimental measurements of the thin films of AlQ_3_-TCNQ, AlQ_3_-DAAq, and ZnQ_2_-DAAq.

To obtain the experimental optical band gap, the Tauc method [56,57] was used, which requires the determination of the absorption coefficient (α) from the transmittance (T) and the thickness (see experimental section) of the films (d) according to the following expression:

A = ln (T/d)

Tauc’s equation was applied to assess the optical band gap of AlQ_3_-TCNQ, AlQ_3_-DAAq, and ZnQ_2_-DAAq films:

(αhv) = B (hv − E_g_)^γ^

**Figure 8:**
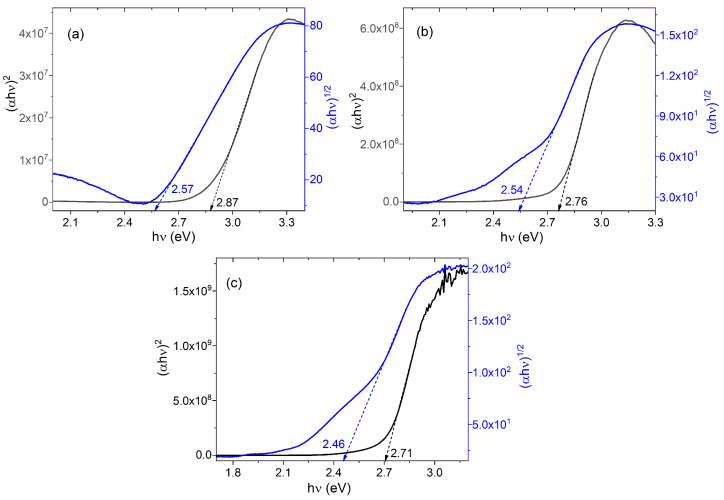
Tauc plots for direct (αhυ)^2^ and indirect (αhυ)^1/2^ transitions of (**a**) AlQ_3_-TCNQ, (**b**) AlQ_3_-DAAq, and (**c**) ZnQ_2_-DAAq films.

In the above expression, the parameter dependent on the inter-band transition probability is denoted by B, the optical band gap is denoted by E_g_, and the index γ defining the nature of the electronic transition responsible for the optical absorption, with γ = 2 and γ = 1/2 for indirect and direct, allowed transitions, respectively [56,57]. The intercept of the extrapolated linear part of the plot of (αhυ)^2^ and (αhυ)^1/2^ against hυ with abscissa in Figure 8 helped to determine the E_g_ value [56,57]. The lowest optical band gaps were obtained for indirect transitions (αhυ)^1/2^ (blue curve), related to amorphous semiconductor films. This is caused by vacuum evaporation, which is the technique used in this work to manufacture the semiconductor films, and where the dispersed heterojunctions in gaseous state are deposited on the substrates that are at room temperature. Thanks to this thermal gradient, the dispersed heterojunctions are deposited in a disordered manner and can generate amorphous films with lower optical band gaps. The lowest band gap was obtained according to the following order: ZnQ_2_-DAAq < AlQ_3_-DAAq < AlQ_3_-TCNQ. This trend is different from those obtained theoretically, although the band gap values, especially for the AlQ_3_-based films, are within the same order of magnitude and are even lower than those calculated theoretically. These experimentally obtained values are also lower than those reported for InQ_3_ films estimated between 2.77 and 2.85 eV [58]; they are also lower than those obtained for GaQ_3_ films of 2.81 eV [59] and for AlQ_3_ films with a band gap of 2.86 eV [53,59]. In the case of hydroxyquinolines, the band gaps vary depending on the component in its dispersed heterojunction or its dopant; for example, those obtained in this work are higher than those reported for ZnQ_2_-TCNQ films of 1.52 eV [47], although they are also lower than the band gap of phosphorus-doped LiQ films, which are in the order of 3.12–3.21 eV [60]. According to these results, the positive effect of the component in its dispersed heterojunction on charge transport is evident, with respect to pristine hydroxyquinoline semiconductors films. For the ZnQ_2_-DAAq, AlQ_3_-DAAq, and AlQ_3_-TCNQ films, it is DAAq that generates the best conduction channels, which is caused by a decrease in the gap between HOMO and LUMO.

**Figure 9:**
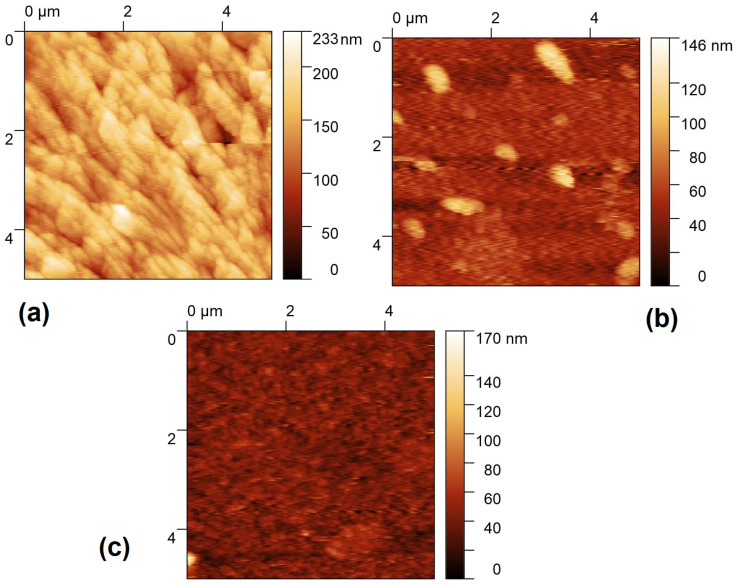
AFM images of (**a**) AlQ_3_-TCNQ, (**b**) AlQ_3_-DAAq, and (**c**) ZnQ_2_-DAAq films.

In the images obtained by AFM and presented in Figure 9, it can be observed that the film completely covers the entire surface of the substrate. Their morphology changes due to the type of dispersed heterojunction. In Table 5, it can be observed that the lowest root mean roughness (RMS) is presented by the ZnQ_2_-DAAq film. It is also important to highlight the mechanical properties of these films. ZnQ_2_-DAAq exhibits the lowest tensile strength (σ) and the lowest Knoop hardness (HK), making it the most susceptible to deformation and wear under service conditions. In contrast, AlQ_3_-TCNQ has a high tensile strength, while AlQ_3_-DAAq demonstrates the highest hardness. Since all films were manufactured under identical conditions, these results suggest that the type of dispersed heterojunction structure plays a crucial role in determining the film’s internal characteristics, including hardness and mechanical resistance. Hardness reflects a material’s ability to resist penetration, whereas mechanical strength determines its capacity to withstand applied loads.

**Figure 10:**
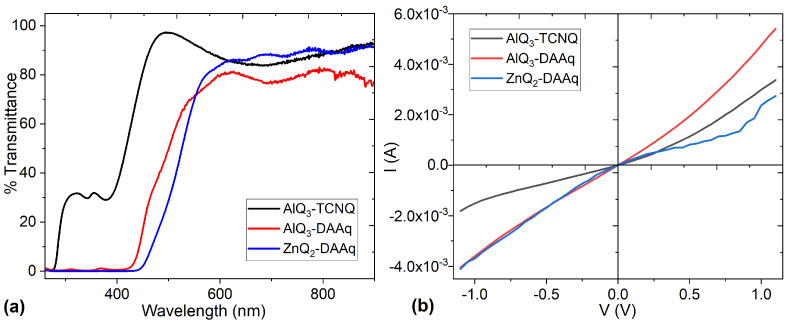
(**a**) Transmittance and (**b**) current–voltage behavior for AlQ_3_-TCNQ, AlQ_3_-DAAq, and ZnQ_2_-DAAq films.

Figure 10a shows the transmittance of the AlQ_3_-TCNQ, AlQ_3_-DAAq, and ZnQ_2_-DAAq films, and it is observed that at λ > 490 nm, the AlQ_3_-TCNQ film exhibits transmittances above 80%, while the other two films have transmittances above 76% at λ > 615 nm. Due to their high transmittance, these films can be used as transparent anodes in organic solar cells, transparent transistors, or photodiode displays and could also be used in smart windows, to mention a few applications.

Finally, to evaluate the current–voltage (I–V) behavior of AlQ_3_-TCNQ, AlQ_3_-DAAq, and ZnQ_2_-DAAq films, single-layer devices with the following conformation were prepared: PET/ITO/dispersed heterojunction/Ag. Maintaining the voltage range from −1.1 V to +1.1 V and under natural illumination conditions, the electric current transported in each device was evaluated, and the results are shown in the curves in Figure 10b. It is observed that the devices with the AlQ_3_-TCNQ and AlQ_3_-DAAq films show an ambipolar behavior, in which, regardless of the polarity of the ITO and Ag electrodes, the current is transported symmetrically following an ohmic behavior. On the other hand, the device with the ZnQ_2_-DAAq film does not present ambipolar behavior because apparently there is a saturation of charges in certain areas of the film, which means that in the positive direction, the current does not flow continuously. This device is due to the above; the one that carries the least electric current and the device with the AlQ_3_-DAAq film is where the most current circulates. It is interesting how, although this film does not have the lowest band gap either theoretically or experimentally, it is the one that presents the best behavior. Regarding its morphology, it is not the film with the least roughness. However, it is the one that transports the most electrical charges with ambipolar behavior, which gives it the potential to be used in organic devices.

## 4. Conclusions

This study examines dispersed heterojunctions formed by AlQ_3_ and ZnQ_2_ in combination with TCNQ and DAAq. The structural calculations were performed iteratively using the B3PW91/6-31G** method, incorporating the GD3 dispersion correction in the second iteration. These organic semiconductors were deposited as thin films and characterized in terms of their morphology, micromechanical properties—including tensile strength and Knoop hardness—as well as their optical and electrical behavior. The dominant and essential property for organic electronics depends on the specific type of dispersed heterojunction. The AlQ_3_-based heterojunctions exhibit the highest tensile strength, hardness, transmittance, and electrical conductivity. Conversely, the ZnQ_2_-based heterojunction presents the lowest band gap. While AlQ_3_ films appear to have the greatest potential for applications in organic electronics, it is important to note that ZnQ_2_-based films exhibit properties closely resembling those of their aluminum counterparts.

## Figures and Tables

**Table 1:** Hydrogen interactions calculated using the B3PW91/6-31G** method. The Wiberg bond index and distances correspond to each interaction.

Interaction (TCNQ/DAAq)·Q_n_(Al/Zn)	Wiberg Index	Distances [Å]
AlQ_3_-TCNQ		
C-H···O-Al (d_1_)	0.016	2.20
C-H···O-Al (d_2_)	0.005	2.71
C=N···H-C (d_3_)	0.004	2.52
AlQ_3_-DAAq		
C=O···H-C (d_1_)	0.003	2.62
C=O···H-C (d_2_)	0.003	2.64
N-H···C (d_3_)	0.005	2.73
ZnQ_2_-DAAq		
N-H···O-Zn (d_1_)	0.024	2.07
N-H···O-Zn (d_2_)	0.021	2.12

**Table 2:** Hydrogen interactions calculated using the B3PW91-GD3/6-31G** method and Wiberg bond index and distances corresponding to each interaction.

Interaction (TCNQ/DAAq)·Q_n_ (Al/Zn)	Wiberg Index	Distances [Å]
AlQ_3_-TCNQ-GD3		
C-H···O-Al (d_1_)	0.003	2.42
C-H···O-Al (d_2_)	0.0007	2.89
C=N···H-C (d_3_)	0.004	2.63
C=N···H-C (d_4_)	0.005	2.64
H-C···O-Al (d_5_)	0.006	2.97
C=N···C (d_6_)	0.002	3.50
C-H···H-C	0.0004	2.41
AlQ_3_-DAAq-GD3		
N-H···O-Al (d_1_)	0.040	1.90
N-H···O-Al (d_2_)	0.0006	3.03
Ring···Ring (d_3_)	-	3.26
ZnQ_2_-DAAq-GD3		
N-H···O-Zn (d_1_)	0.026	2.00
N-H···O-Zn (d_2_)	0.003	2.74
Ring···Ring (d_3_)	-	3.31

**Table 3:** IR frequencies and their assignment for AlQ_3_-TCNQ, AlQ_3_-DAAq, and ZnQ_2_-DAAq pellets and films.

Sample	C=C (cm^−1^)	C-N Quinoline (cm^−1^)	C-O (cm^−1^)	In Plane-Ring Deformation (cm^−1^)	O-M (cm^−1^)	-C≡N (TCNQ) (cm^−1^)	C=O (DAAq) (cm^−1^)	N-H (DAAq) (cm^−1^)	C-H (DAAq) (cm^−1^)
AlQ_3_ KBr pellet	1605	1581, 1329	1471, 1281	805, 751	648				
ZnQ_2_ KBr pellet	1610	1582, 1326	1487, 1282	803, 735	652				
TCNQ KBr pellet						2222			
DAAq KBr pellet							1664	3334, 3207, 1497	3059
AlQ_3_-TCNQ, KBr pellet	1603	1575, 1318	1464, 1270	790, 737	647	2194			
AlQ_3_-TCNQ, film	1602	1575, 1323	1463, 1277	789, 732	646	2214			
AlQ_3_-DAAq KBr pellet	1605	1577, 1328	1476, 1285	787, 749	649		1657	3334, 3204, 1494	3047
AlQ_3_-DAAq film	1608	1580, 1323	1471, 1280	790, 750	645		1660	3340, 3210, 1499	3049
ZnQ_2_-DAAq KBr pellet	1607	1580, 1323	1462, 1275	790, 732	647		1658	3336, 3207, 1492	3041
ZnQ_2_-DAAq film	1609	1578, 1463	1463, 1278	790, 732	648		1660	3348, 3219, 1494	3048

**Table 4:** Schematization of the HOMO and LUMO molecular orbitals, their respective energies, and the HOMO–LUMO gap.

	HOMO (eV)	LUMO (eV)	ΔE (eV)
AlQ_3_-TCNQ	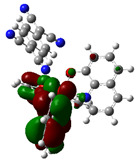 −5.5155	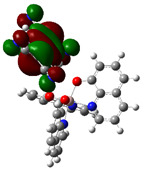 −4.2755	1.24
AlQ_3_-DAAq	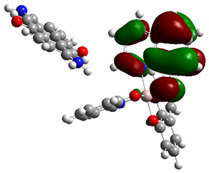 −5.1998	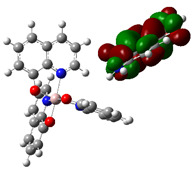 −2.4991	2.70
ZnQ_2_-DAAq	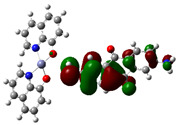 −5.0673	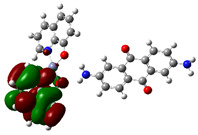 −2.1789	2.89
AlQ_3_-TCNQ-GD3	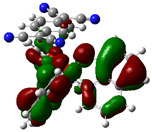 −6.6287	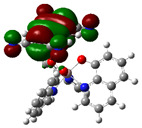 −4.0091	1.62
AlQ_3_-DAAq-GD3	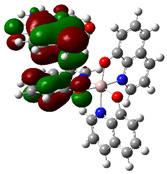 −5.2469	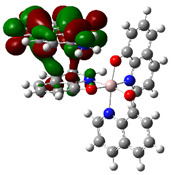 −2.2692	2.98
ZnQ_2-_DAAq-GD3	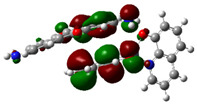 −5.1628	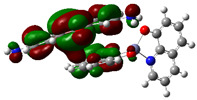 −2.1987	2.97

**Table 5:** Root mean roughness (RMS), tensile strength (σ), and Knoop hardness (HK) of AlQ_3_-TCNQ, AlQ_3_-DAAq, and ZnQ_2_-DAAq films.

Film	Thickness (μm)	RMS (nm)	σ (Pa)	HK
AlQ_3_-TCNQ	6.2	37.65	2.97 × 10^−3^	14.41
AlQ_3_-DAAq	6.6	27.06	1.52 × 10^−4^	18.13
ZnQ_2_-DAAq	6.3	11.97	1.04 × 10^−6^	0.0002

## Data Availability

Data are contained within the article.

## Supplementary Materials

The following supporting information can be downloaded at: https://www.mdpi.com/article/10.3390/molecules30091896/s1, Figure S1: From left to right, comparison between experimental and theoretical absorbance spectra for (a) AlQ_3_-TCNQ, (b) AlQ_3_-DAAq, and (c) ZnQ_2_-DAAq using TD-DFT PBE/TZP method. To build each theoretical spectrum, a Gaussian broadening of 30 nm was applied; Table S1: Singlet–singlet transitions for AlQ_3_-TCNQ-GD3. Experimental peaks, theoretical transition energy, oscillator strength, molecular orbital transitions, and their weights; Table S2: Singlet–singlet transitions for AlQ_3_-DAAq-GD3. Experimental peaks, theoretical transition energy, oscillator strength, molecular orbital transitions, and their weights; Table S3: Singlet–singlet transitions for ZnQ_2_-DAAq-GD3. Experimental peaks, theoretical transition energy, oscillator strength, molecular orbital transitions, and their weights.
