# Supplementary material for: Optimization of Zinc and Aluminum Hydroxyquinolines for Applications as Semiconductors in Molecular Electronics
Source: Molecules. 2025 Apr 24;30(9):1896. doi: 10.3390/molecules30091896 (PMC12074348; doi:10.3390/molecules30091896)
Supplement: Supplementary file 1 [file molecules-30-01896-s001.zip › molecules-3536640-supplementary.pdf]

Supporting Information for Optimization of Zinc and Aluminum Hydroxyquinolines: A Study on Their Doping for Applications as Semiconductors in Molecular Electronics

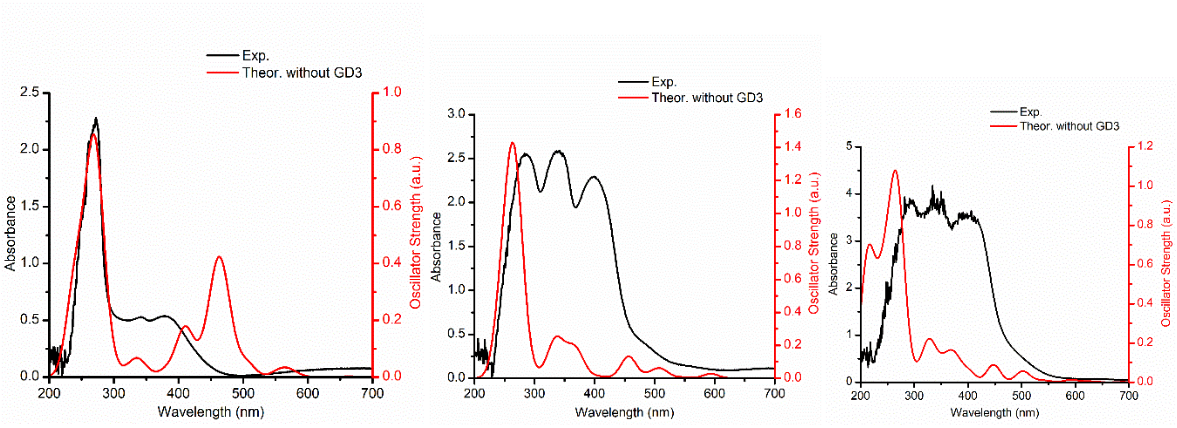

(a) (b) (c)

Figure S1. From left to right, comparison between experimental and theoretical absorbance spectra for (a) AlQ_3-TCNQ, (b) AlQ_3-DAAq, (c) ZnQ_2-DAAq using TD-DFT PBE/TZP method. To build each theoretical spectrum, a gaussian broadening of 30 nm was applied.

Table S1. Singlet-Singlet transitions for AlQ_3-TCNQ-GD3. Experimental peaks, theoretical transition energy, oscillator strength, molecular orbital transitions, and their weights.

**Peaks**	**Theor. (nm)**	**Transition energy (nm)**	**f (a.u.)**	**MO Transitions Occup. Orbital →Unoccup. Orbital**	**Weight (%)**
		234.6	0.049	HOMO-5 → LUMO+10	12.3
a	271			HOMO-2 → LUMO+12	10.8
		265.6	0.066	HOMO → LUMO+15	69.8
		266.6	0.111	HOMO → LUMO+15	23.2
				HOMO-2 → LUMO+13	21.6
		269.1	0.055	HOMO-2 → LUMO+13	16.2
				HOMO-11 → LUMO+2	14.4
		271.7	0.086	HOMO-6 → LUMO+1	12.0
				HOMO-33 → LUMO	11.2
				HOMO-2 → LUMO+13	8.0
		277.1	0.044	HOMO → LUMO+12	23.9
				HOMO-12 → LUMO+2	22.5
b	340	339.7	0.019	HOMO → LUMO+8	27.6
				HOMO-4 → LUMO+2	14.8
c	376	374.2	0.057	HOMO-2 → LUMO+6	46.9
		376.8	0.657	HOMO-2 → LUMO+5	43.9

Table S2. Singlet-Singlet transitions for AlQ$_3$-DAAq-GD3. Experimental peaks, theoretical transition energy, oscillator strength, molecular orbital transitions, and their weights.

Peaks	Theor. (nm)	Transition energy (nm)	f (a.u.)	MO Transitions Occup. Orbital →Unoccup. Orbital	Weight (%)
		240.5	0.056	HOMO → LUMO+17	42.6
a	265			HOMO-5 → LUMO+13	27.8
		262.9	0.093	HOMO-2 → LUMO+14	41.0
				HOMO-17 → LUMO+3	13.5
		264.9	0.217	HOMO-17 → LUMO+3	15.3
				HOMO-16 → LUMO+3	15.3
		269.4	0.103	HOMO-1 → LUMO+4	22.4
				HOMO-12 → LUMO+1	11.7
				HOMO-11 → LUMO+2	10.1
		272.9	0.048	HOMO-2 → LUMO+11	9.0
				HOMO-8 → LUMO+3	8.7
				HOMO-16 → LUMO+3	8.4
		287.8	0.037	HOMO-7 → LUMO+4	33.0
				HOMO-8 → LUMO+4	28.4
b	328	322.3	0.117	HOMO-9 → LUMO	29.7
				HOMO-5 → LUMO+4	27.0
		331.6	0.019	HOMO → LUMO+7	29.8
				HOMO-14 → LUMO	16.8
		337.8	0.018	HOMO-1 → LUMO+6	26.9
				HOMO-1 → LUMO+7	15.7
		341.4	0.017	HOMO-5 → LUMO+4	31.7
				HOMO-14 → LUMO+5	17.3
c	392	388.7	0.025	HOMO-1 → LUMO+5	40.6
		390.1	0.090	HOMO-1 → LUMO+5	25.3
				HOMO-2 → LUMO+4	24.3
		396.0	0.056	HOMO-2 → LUMO+5	38.4
				HOMO-2 → LUMO+4	17.5
d	473	448.8	0.040	HOMO → LUMO+4	47.2
				HOMO-5 → LUMO	41.0
		505.7	0.021	HOMO-1 → LUMO+2	60.6

Table S3. Singlet-Singlet transitions for ZnQ_2-DAAq-GD3. Experimental peaks, theoretical transition energy, oscillator strength, molecular orbital transitions, and their weights.

Peaks	Teor. (nm)	Transition energy (nm)	f (a.u.)	MO Transitions Occup. Orbital →Unoccup. Orbital	Weight (%)
a	261	245.9	0.080	HOMO-4 → LUMO+10	22.0
				HOMO-19 → LUMO	21.8
		265.3	0.143	HOMO-5 → LUMO+6	11.7
				HOMO-9 → LUMO+3	10.6
				HOMO-10 → LUMO+1	8.8
		266.5	0.126	HOMO-10 → LUMO+1	37.5
				HOMO-11 → LUMO+3	15.0
		271.1	0.064	HOMO-1 → LUMO+10	31.1
				HOMO-14 → LUMO	11.1
		277.1	0.040	HOMO-1 → LUMO+9	54.1
b	328	324.8	0.136	HOMO-4 → LUMO+3	38.3
				HOMO-9 → LUMO	24.1
		326.5	0.022	HOMO-8 → LUMO+2	27.6
				HOMO → LUMO+7	18.4
				HOMO-2 → LUMO+5	16.2
		338.5	0.015	HOMO-2 → LUMO+4	40.2
				HOMO-4 → LUMO+4	17.9
c	375	359.0	0.035	HOMO-8 → LUMO	65.8
		376.0	0.115	HOMO-2 → LUMO+3	46.4
				HOMO-1 → LUMO+3	26.4
d	483	449.2	0.063	HOMO-4 → LUMO+3	80.5
		491.5	0.033	HOMO-2 → LUMO+1	71.6
